# BAY 87-2243, a highly potent and selective inhibitor of hypoxia-induced gene activation has antitumor activities by inhibition of mitochondrial complex I

**DOI:** 10.1002/cam4.112

**Published:** 2013-08-20

**Authors:** Peter Ellinghaus, Iring Heisler, Kerstin Unterschemmann, Michael Haerter, Hartmut Beck, Susanne Greschat, Alexander Ehrmann, Holger Summer, Ingo Flamme, Felix Oehme, Karlheinz Thierauch, Martin Michels, Holger Hess-Stumpp, Karl Ziegelbauer

**Affiliations:** 1 Bayer Pharma AG, Global Drug DiscoveryWuppertal, Germany; 2 Bayer Pharma AG, Global Drug DiscoveryBerlin, Germany

**Keywords:** Antitumor activity, hypoxia, hypoxia-inducible factor-1, mitochondrial complex 1

## Abstract

The activation of the transcription factor hypoxia-inducible factor-1 (HIF-1) plays an essential role in tumor development, tumor progression, and resistance to chemo- and radiotherapy. In order to identify compounds targeting the HIF pathway, a small molecule library was screened using a luciferase-driven HIF-1 reporter cell line under hypoxia. The high-throughput screening led to the identification of a class of aminoalkyl-substituted compounds that inhibited hypoxia-induced HIF-1 target gene expression in human lung cancer cell lines at low nanomolar concentrations. Lead structure BAY 87-2243 was found to inhibit HIF-1α and HIF-2α protein accumulation under hypoxic conditions in non-small cell lung cancer (NSCLC) cell line H460 but had no effect on HIF-1α protein levels induced by the hypoxia mimetics desferrioxamine or cobalt chloride. BAY 87-2243 had no effect on HIF target gene expression levels in RCC4 cells lacking Von Hippel–Lindau (VHL) activity nor did the compound affect the activity of HIF prolyl hydroxylase-2. Antitumor activity of BAY 87-2243, suppression of HIF-1α protein levels, and reduction of HIF-1 target gene expression in vivo were demonstrated in a H460 xenograft model. BAY 87-2243 did not inhibit cell proliferation under standard conditions. However under glucose depletion, a condition favoring mitochondrial ATP generation as energy source, BAY 87-2243 inhibited cell proliferation in the nanomolar range. Further experiments revealed that BAY 87-2243 inhibits mitochondrial complex I activity but has no effect on complex III activity. Interference with mitochondrial function to reduce hypoxia-induced HIF-1 activity in tumors might be an interesting therapeutic approach to overcome chemo- and radiotherapy-resistance of hypoxic tumors.

## Introduction

Hypoxia is a hallmark of solid tumors^1^. Adaption to hypoxia is critical for tumor survival and growth and is mediated in large part by transcriptional activation of genes that facilitate short-term adaptive mechanisms, for example, increased vascular permeability, vasodilation, glucose transport, switch to anaerobic metabolism, as well as long-term adaptive mechanisms, for example, angiogenesis^2^. The response of mammalian cells to hypoxia is mediated by a family of transcription factors known as hypoxia-inducible factors (HIF). HIF-1 is a heterodimer consisting of the constitutively expressed HIF-1β subunit (also known as aryl hydrocarbon receptor nuclear translocator) and an oxygen-regulated alpha subunit. There are three alpha-isoforms: HIF-1α is nearly ubiquitously expressed whereas HIF-2α and HIF-3α show a more restricted expression pattern. HIF-1α and HIF-1β associate in the cytosol prior to transport to the nucleus where they bind to hypoxia response elements (HREs) in the 3′ and 5′ regions of hypoxia-regulated genes^3^. The HIF-1α and HIF-2α subunits are structurally similar in their DNA binding and dimerization domains but differ in their transactivation domains implying they may have unique target genes. Under normoxia, both HIF-1α and HIF-2α are degraded rapidly through a posttranslational process involving proline hydroxylation in an oxygen-dependent manner targeting HIF proteins to the Von Hippel–Lindau (VHL) ubiquitination complex for final degradation by the proteasome system. Under reduced oxygen pressure proline hydroxylation diminishes, and HIF-1α and HIF-2α accumulate^4^.

HIF-1α overexpression is observed in >70% of human cancers and is associated with poor patient prognosis and resistance to radio- and chemotherapy^5^,^6^. Vice versa, HIF-1α “loss-of-function” results in inhibition of tumor growth in experimental xenograft studies with varying degree^7^,^8^. Because of the importance of HIF-1 in tumor development and progression, many efforts have been devoted to identify small molecule HIF-1 inhibitors for cancer therapy in pharmaceutical research, for example, as described in^9^. However, many of these compounds have activities other than HIF inhibition that may contribute to their antitumor activity and lack the desired pharmacokinetic properties or safety profiles required for a useful pharmaceutical agent. Here, we identified in a small molecule library screen a series of compounds that potently inhibited HIF-1α protein accumulation and thus HIF-1 target gene expression at low nmol/L concentrations without affecting the expression levels of genes not regulated by HIF-1/hypoxia or inhibiting cellular proliferation under normoxia. The lead compound BAY 87-2243 showed dose-dependent in vivo antitumor efficacy in the H460 lung tumor xenograft model accompanied by a suppression of HIF-1α protein and HIF-1 target genes without any signs of toxicity or body weight loss. Further mode-of-action analyses revealed that BAY 87-2243 exerts its effect on the HIF pathway by blocking mitochondrial complex I activity and thereby reducing HIF protein levels under hypoxia.

## Material and Methods

### Bay 87-2243

**Figure 1:**
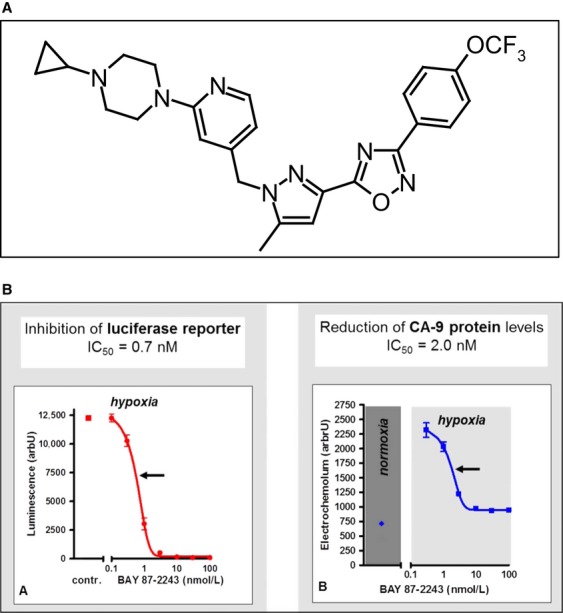
(A) Chemical structure of BAY 87-2243. (B) BAY 87-2243 suppresses hypoxia-induced reporter gene activity (left), and CA9 protein levels in vitro in HCT-116 cell lysates (right). HCT-116 cells were stably transfected with 4×VEGF promoter-derived HIF response element in front of a luciferase reporter gene and incubated for 24 h under hypoxia (1% *p*O_2_). IC_50_ values were calculated by GraphPad Prism. VEGF, vascular endothelial growth factor; HIF, hypoxia-inducible factor.

1-Cyclopropyl-4-{4-[(5-methyl-3-{3-[4-(trifluoromethoxy)phenyl]-1,2,4-oxadiazol-5-yl}-1H-pyrazol-1-yl)methyl]pyri-din-2-yl}piperazine (BAY 87-2243, Bayer Pharma AG, Wuppertal, Germany) was derived from an initial luciferase screening hit after intensive medicinal chemistry optimization for potency and improved physicochemical properties (Fig. 1A). BAY 87-2243 was synthesized according to the processes described in the International Patent Application Publication Number WO 2010/054763, in particular as described in example 65. For in vitro studies, BAY 87-2243 was prepared as a 10 mmol/L stock solution in dimethyl sulfoxide (DMSO) and diluted in the relevant assay media. For in vivo studies, BAY 87-2243 was formulated in a 1% (v/v) solution of ethanol/solutol/water (10/40/50%). Animals were given BAY 87-2243 (0.5, 1, 2, and 4 mg/kg) or vehicle control once daily by oral gavage.

### Cell culture

The human colon carcinoma cell line HCT-116, the human large cell lung cancer cell line H460, the human non-small cell lung cancer (NSCLC) cell line H1299, the human dopaminergic neuronal cell line SH-SY5Y, and the human prostate cancer cell line PC-3 were obtained from American Type Culture Collection and not cultured for more than 6 months before conducting the experiments described here. Renal cell carcinoma cell line RCC4 stably transfected with either an empty expression vector pcDNA3 or with pcDNA3-VHL were obtained from the European Collection of Cell Cultures (ECACC). All cell lines were routinely grown in standard medium recommended by the supplier and supplemented with 10% (v/v) fetal calf serum (FCS, Life technologies, Darmstadt, Germany) if not indicated otherwise.

### Cellular assays

For high-throughput screening of a small molecule library consisting of ∼830,000 compounds, HCT-116 cells were stably transfected with a vector containing a luciferase reporter system coupled four times to the HRE from human vascular endothelial growth factor (VEGF) promoter (HCT 116-4xVEGF-Luc). Cells were plated at 3 × 10E4 cells/well and incubated overnight before test compounds (5 mmol/L in DMSO) were added and plates were placed in a hypoxic chamber for 16 h at 1% *p*O_2_. Results are given as luminescence counts in arbitrary units after subtraction of baseline levels from normoxic, nontreated controls. For the measurement of cellular complex I activity, H1299 cells were cotransfected with a pcDNA3 vector encoding for *Pyrearinus termitilluminans* larval click beetle luciferase^10^. Clones (H1299tluc) showing high luminescence and dose-dependent rotenone sensitivity were subcloned and then used for further in depth analysis of cellular complex I activity by luminescence measurements. In brief, H1299tluc cells (1500/well) were seeded into white 384 well plates. After 2 days in culturing in Dulbecco's modified eagle medium (DMEM) without glucose, but supplemented with 11 mmol/L galactose, 10 μL of a luciferin/inhibitor mixture (150 μmol/L d-luciferin, 0.4% DMSO final concentration in Tyrode) was added to each well and incubated for 1 h at 37°C. Luminescence measurements were performed with an in house developed plate reader. After this measurement 20 μL succinate (0.67 mol/L, pH 5.3 in Tyrode, final concentration 25 mmol/L) was added. The plate was then incubated for another 1 h at room temperature before the second measurement was performed. H1299tluc cells expressing NADH-Q-Oxidoreductase from *Saccharomyces cerevisiae* (NDI1) were generated by transfection with a pcDNA3 vector encoding for NDI1 under control of a cytomegaly virus (CMV) promoter and a C-terminal HA-tag using PiggyBac transposon-mediated gene transfer^11^. Selection for positive clones was performed by cultivation in the presence of 20 nmol/L rotenone in DMEM medium with 11.2 mmol/L glucose. Rotenone insensitive clones with high luminescence were used as described above. Luciferase activity is given in % of DMSO-treated cells. To evaluate the cytotoxicity of BAY 87-2243, 2.000 cells of the respective cell lines were seeded in 96-well plates and cultured in the appropriate growth medium containing 10% FCS. BAY 87-2243 at various concentrations was added at 24 h after seeding for additional 48 h and cell viability was determined using Cell Titer Glow Assay (Promega, Heidelberg, Germany).

### Quantification of CA9 protein

HCT 116-4xVEGF-Luc cells were seeded at 3 × 10E4 cells/well in 96-well plates and incubated overnight at 37°C in a humidified incubator containing 5% CO_2_ under normal oxygen levels before shifting hypoxic conditions (1% *p*O_2_, 24 h) in the absence or presence of various concentrations of BAY 87-2243. Protein expression levels of the HIF target gene carbonic anhydrase 9 (CA9) in cell lysates was quantified using MN/CAIX enzyme linked immunosorbent assay (Siemens, cat# 06490025).

### Western blot for HIF-1α and HIF-2α

H460 cells and RCC4 cells transfected with empty pcDNA vector were grown to 70% confluency and incubated with various concentrations of BAY 87-2243 under either normoxia or hypoxia (1% *p*O_2_) for 16 h. Cytosolic extracts and nuclear extracts were prepared using NE-PER kit from Pierce [Schwerte, Germany; cat# 22660] and subjected to Western Blot analyses. HIF-1α protein was detected by a polyclonal antibody from BD Biosciences Pharmingen [cat# 610958], HIF-2α by a monoclonal antibody from Novus Biologicals [Heidelberg, Germany; cat# NB100-132]. As lane loading controls, polyclonal β-Actin antibody from Cell Signaling [Frankfurt, Germany; cat# 4967] was used; for either cytosolic or whole cell extracts a polyclonal Lamin B1 antibody from Abcam [Cambridge, U.K.; cat# ab16048] was used for nuclear extracts. Detection was performed with horseradish peroxidase-labeled anti-rabbit Ig1 antibody from Amersham [Freiburg, Germany; cat# NA934VS]. Prolyl hydroxylase inhibitor (PHDI) was purchased from Selleckchem, Munich, Germany.

### Real-time PCR

Total RNA from H460 xenograft tumors was isolated by phenol/chloroform extraction. Total RNA from cell lines was isolated using RNeasy system (Qiagen, Hilden, Germany). cDNA synthesis and subsequent Real-time polymerase chain reaction (PCR) using 6-carboxyfluorescein/Carboxytetramethylrhodamine-labeled probes were carried out as described in^12^. Results are expressed in arbitrary units after normalization of raw data to cytosolic beta-actin levels from each sample as described in^13^. The sequences of primer/probes designed by using PrimerExpress software (Applied Biosystems) are given in Table S2.

### Microarray experiments

Hybridization of Affymetrix GeneChip Human Gene 1.0 ST (High Wycombe, U.K.) arrays was carried out as described in^14^. In brief, 1 μg of total RNA from H460 cells was reverse transcribed into cDNA and amplified by T7 RNA polymerase. Quality of resulting cRNA was assessed by a 2100 Bioanalyzer (Agilent). Each microarray was washed and stained with strepatavidin-phycoerythrin and then scanned with Affymetrix Scanner 3000 according to the technical manual of Affymetrix. Fluorescence intensities of scanned images were corrected for background noise and evaluated using the Affymetrix Microarray Analysis Suite (MAS 5.0) software. Further statistical analysis was performed with expressionist software (Genedata, Basel, Switzerland). The data discussed in this publication have been deposited in National Center for Biotechnology Information Gene Expression Omnibus (GEO, http://www.ncbi.nlm.nih.gov/geo/) and are accessible through GEO Series accession number GSE42791.

### In vivo tumor study

Tumor xenograft experiment was carried out on female immune-deficient, athymic NMRI nude mice (Taconic M&B), aged 7–9 weeks, weighing 20–25 g in full accordance with the Interdisciplinary Principles and Guidelines for the Use of Animals in Research, Marketing and Education issued by the New York Academy of Sciences' Ad Hoc Committee on Animal Research. The lung carcinoma xenograft mouse model was established by subcutaneous injection into the right flank with 0.1 mL H460 tumor cells (1.5 × 10E6) mixed 1:1 with Matrigel (Becton Dickinson, Mannheim, Germany). Mice were randomized into control and treatment groups when tumors reached a size of more than 40 mm^2^. Body weight was monitored as a measure for treatment-related, acute toxicity. Tumor area (measured by caliper) or tumor weight (measured when mice were sacrificed 21 days after cell injection) was calculated by the formula 100−100 × (tumor weight/area of treatment group)/(tumor weight/area of vehicle group). Tumor Statistical analysis was performed using the one-way analysis of variance (ANOVA) (SigmaStat software, Erkrath, Germany).

### Murine plasma pharmacokinetic analyses

Plasma concentrations of unchanged BAY 87-2243 were determined by liquid chromatography coupled to a tandem mass spectrometer (LC-MS/MS). Briefly, murine plasma was centrifuged and subsequently precipitated by addition of acetonitrile and an internal standard. The supernatants were subjected to high-performance LC (Agilent 1100 LC Quatronary Pump, Agilent Technologies, Waldbronn, Germany) connected to a MS/MS (API 3000, Applied Biosystems, Darmstadt, Germany) via a Turbo Ion Spray interface.

### Transfection with small interfering RNA for EGLN1

Small interfering RNA (siRNA) targeting Egl nine homolog 1 (EGLN1) and negative controls (scrambled siRNA) were purchased from Qiagen (Germany). H460 cells were grown to 70% confluency and transfected with siRNA using Effectene (Qiagen) for 48 h in Roswell Park Memorial Institute medium containing 10% FCS. Efficacy of EGLN1-silencing was confirmed by Real-time PCR quantification of EGLN1 mRNA levels compared to scrambled siRNA-transfected cells. Subsequently, cells were incubated for 24 h with various concentrations of BAY 87-2243 under normoxia before HIF target gene expression levels were assessed by real-time PCR.

### Prolyl hydroxylase activity assay

The influence of the test compound on prolyl hydroxylase 2 (PHD2) activity was assayed as described previously^15^ with some modifications: Recombinant human PHD2 was purified from Sf9 cell lysates and used for hydroxylation of biotinylated HIF-1α 556-574 peptide coated on NeutrAvidin plates (Pierce). Hydoxylated peptide was quantified after incubation with purified Von-Hippel-Lindau L-Elongin B-Elongin Complex complex labeled with europium (DELFIA labeling reagent, Perkin-Elmer, Waltham, MA) and addition of enhancer solution (Perkin-Elmer) by measuring time-resolved fluorescence with a Tecan infinite M200 plate reader.

### Measurement of mitochondrial oxygen consumption

Mitochondrial oxygen consumption was determined using the MitoXpress™-Xtra assay from Luxcel at Mitologics, Paris, France. Therefore, mitochondria were isolated from human prostate adenocarcinoma PC-3 cell line. To ensure quality of mitochondrial preparations, samples were subjected to various assays for integrity and functionality including forward scattter/side scatter FACScan (BD Bioscience, Mannheim, Germany) analysis in the presence or absence of Mitotracker™ green (DYm insensitive, Invitrogen, Darmstadt, Germany) and Mitotracker™ red (DYm sensitive). Isolated mitochondria were incubated in presence and absence of BAY 87-2243 with glutamate (12.5 mmol/L), malate (5 mmol/L), ADP (1.65 mmol/L) and a fluorescent dye sensitive to oxygen (LUX-MitoXpress; LUXCEL; 1 μmol/L) with or without the known complex I inhibitor rotenone (2 μmol/L). Oxygen consumption was measured by using Tecan Infinite 200 system (380 nm/650 nm). Complex I inhibition in % was calculated comparing maximal stimulation of respiration by glutamate, malate and ADP only (=100%) and the inhibitory effect of 2 μmol/L rotenone (=0%).

### Statistical analysis

Results are either representative or average of at least three independent experiments done. Statistical analysis was performed using ANOVA test and *t* test (GraphPad Prism).

## Results

### BAY 87-2243 inhibits HIF-1 reporter gene activity and CA9 protein expression

To test whether BAY 87-2243 inhibited HIF-1 activity, HCT-116 cells were stably transfected with pGL2-TK-HRE, containing the luciferase reporter gene under control of four copies of a HRE derived from human VEGF promoter. Hypoxia increased HRE-dependent luciferase expression more than 100-fold relative to HCT-116luc cells cultured under normoxic conditions. BAY 87-2243 inhibited luciferase activity with a calculated IC_50_ value of ∼0.7 nmol/L (Fig. 1B, left). Hypoxic induction of the HIF target gene CA9 on protein level in HCT116luc cells was inhibited by BAY 87-2243 with an IC_50_ value of ∼2.0 nmol/L (Fig. 1B, right).

### BAY 87-2243 suppresses HIF target gene expression in hypoxic lung cancer cells

**Figure 2:**
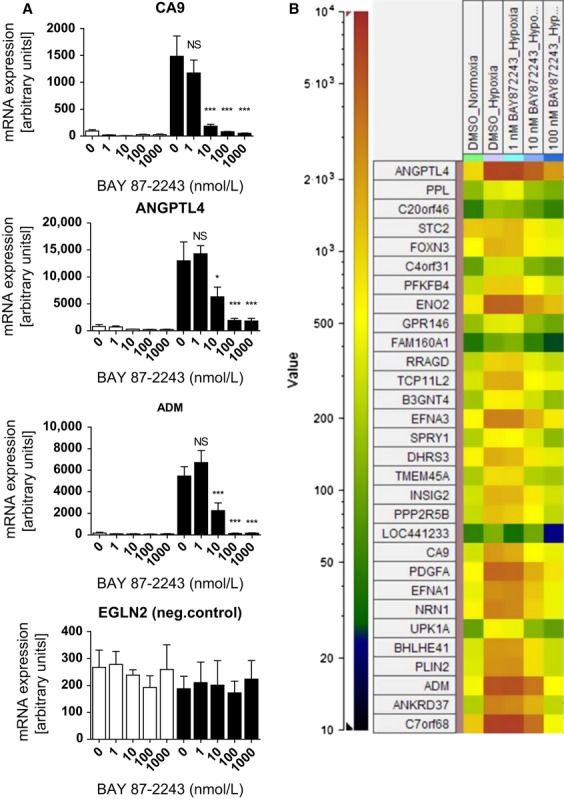
BAY 87-2243 inhibits HIF target gene expression in vitro. (A) H460 cells were cultured for 16 h under normoxia or hypoxia (1% *p*O_2_) in the absence or presence of various concentrations of BAY 87-2243. MRNA expression levels of the HIF target genes CA9, ADM, ANGPTL4 and the negative control gene EGLN2 were quantified by RT-PCR. (B) RNA from vehicle-treated normoxic H460 cells and from hypoxic H460 cells incubated with 1, 10, and 100 nmol/L BAY 87-2243, respectively, was subjected to microarray hybridization. A heatmap shows the mRNA signal intensity of those 30 genes that were most strongly suppressed by 100 nmol/L BAY 87-2243 in hypoxic H460 cells compared to DMSO-treated hypoxic H460 cells. HIF, hypoxia-inducible factor; RT-PCR, real-time polymerase chain reaction.

To evaluate dose dependency of BAY 87-2243 on HIF-1 transcriptional activity, H460 cells were cultured under normoxia and hypoxia (16 h, 1% *p*O_2_) with various concentrations of BAY 87-2243 ranging from 1 to 1000 nmol/L, and the mRNA level of the HIF-1 target genes CA9, adrenomedullin (ADM), and angiopoietin-like protein-4 (ANGPTL4) was quantified by real-time PCR. EGLN2, a PHD known to be expressed independent of the oxygen supply served as a negative control. BAY 87-2243 suppressed HIF-1 target gene expression dose dependently under hypoxia, but a weak reduction of baseline HIF-1 target gene mRNA expression levels was also observed under normoxia. Notably, expression of EGLN2 was not affected by BAY 87-2243 treatment neither under normoxia nor under hypoxia (Fig. 2A). Additional negative controls (genes not regulated by hypoxia) were evaluated by real-time PCR after incubation of normoxic and hypoxic H460 cells with BAY 87-2243 at concentrations up to 10 μmol/L. No signs of unspecific transcription inhibition were observed even at such high dosages (Fig. S1).

Specificity of BAY 87-2243 for the suppression of HIF-1-mediated gene transcription on a genome-wide scale was evaluated by microarray hybridizations using Affymetrix whole genome 133Av2 arrays. RNA from normoxic H460 cells and from hypoxic H460 cells incubated with 1, 10, and 100 nmol/L BAY 87-2243, respectively, was subjected to array hybridization. Of those 30 genes that were most strongly suppressed by 100 nmol/L BAY 87-2243 in hypoxic H460 cells compared to DMSO-treated hypoxic H460 cells, virtually all of them are induced by prior hypoxia and most of these genes have been described in the literature as HIF-1 target genes (Fig. 2B), including ANGPTL4, ADM, and CA9.

### BAY 87-2243 inhibits HIF-1α protein accumulation in hypoxic lung cancer cells but has no effect on HIF-1α protein levels induced by hypoxia mimetics

**Figure 3:**
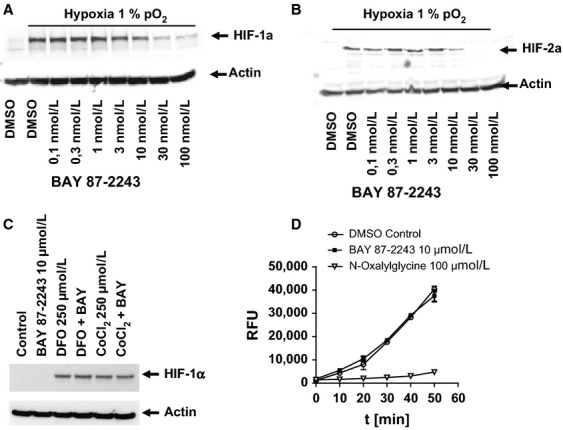
BAY 87-2243 inhibits hypoxia-inducible factor (HIF-1α) and HIF-2α protein accumulation in hypoxic H460 cells H460 under hypoxia but has no effect on HIF-1α protein levels induced by hypoxia mimetics and has no effect on prolyl hydroxylase 2 (PHD2) activity. (A, B) H460 cells were cultured for 16 h under normoxia or hypoxia (1% *p*O_2_) in the absence or presence of various concentrations of BAY 87-2243. HIF-1α (A) and HIF-2α (B) protein levels were assessed by Western Blot in whole cell extracts. β-actin was used as a loading control. (C) H460 cells were cultured for 16 h under normoxia with the PHDs desferrioxamine (DFO) and CoCl_2_ plus/minus BAY 87-2243 before the HIF1α protein levels in cellular extracts were quantified by Western Blot. β-actin was used to as a loading control. (D) Effect of BAY 87-2243 on the recombinant PHD2-mediated hydroxylation of HIF-1α peptide over time was measured in a biochemical assay. Hydoxylated peptide was quantified after incubation with purified VBC complex labeled with europium using fluorescence as a readout. The known PHD inhibitor *N*-oxalylglycine served as a positive control.

To address whether BAY 87-2243 inhibited HIF-1α protein accumulation, H460 cells were incubated with various concentrations of BAY 87-2243 under hypoxia. BAY 87-2243 inhibits HIF-1α and HIF-2α protein accumulation dose dependently in total cellular extracts from hypoxic H460 cells (Fig. 3A and B), but had no effect on HIF-1α protein levels induced by the hypoxia mimetics desferrioxamine of cobalt chloride (Fig. 3C).

### BAY 87-2243 has no effect on PHD-2 activity

To exclude a possible activation of the HIF degrading PHD as the underlying cause for the diminished HIF-1α and HIF-2α protein levels under hypoxia upon BAY 87-2243 treatment, we quantified the amount of hydroxylation of a biotinylated HIF-1α 556-574 peptide by recombinant PHD2 in the absence or presence of BAY 87-2243 over time^15^. BAY 87-2243 at a concentration of 10 μmol/L had no effect on the PHD2 activity whereas the known inhibitor of the PHD activity, *N*-oxalylglycine at 100 μmol/L strongly reduced the hydroxylation of the HIF-1α peptide by PHD2 (Fig. 3D).

### BAY 87-2243 activity depends on intact VHL protein and PHD activity

**Figure 4:**
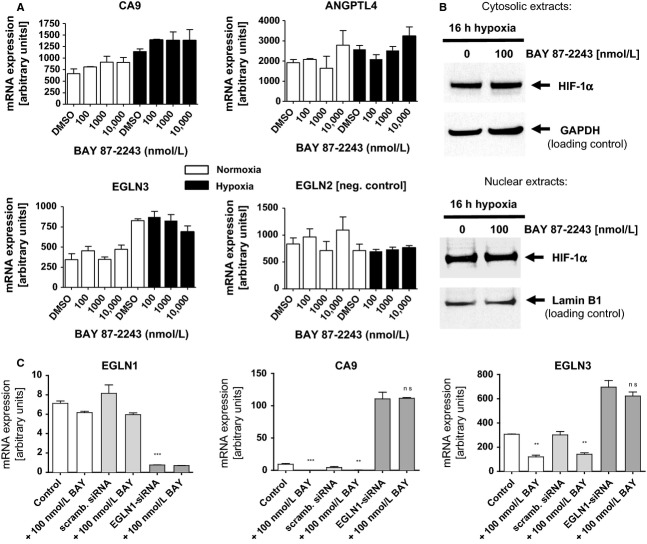
BAY 87-2243 is inactive in RCC4 cells lacking functional VHL protein or in H460 cells silenced for EGLN1. (A) RCC4 cells were incubated under normoxia or hypoxia for 16 h with the indicated concentrations of BAY 87-2243. Expression levels of HIF-1 target genes CA9, ANGPTL4, EGLN3, and of negative control EGLN2 were quantified by real-time PCR. (B) HIF-1α protein levels in hypoxic RCC4 cells treated for 16 h with 100 nmol/L BAY 87-2243 were assessed by Western Blot in cytosolic and nuclear extracts. β-actin was used to as a loading control for cytosolic extracts and Lamin B1 as a loading control for nuclear extracts. (C) H460 cells were transfected with either an EGLN1-silencing RNA or a scrambled control siRNA for 48 h before incubation with 100 nmol/L BAY 87-2243 for additional 24 h under normoxia. RNA was isolated and the expression levels of EGLN1 (to confirm silencing), and the HIF-1 target genes CA9, EGLN3, and VEGF were quantified by real-time PCR. VHL, Von Hippel–Lindau; HIF, hypoxia-inducible factor; VEGF, vascular endothelial growth factor.

HIF-1α steady state is the result of a balance between protein translation and its proteasomal degradation mediated by Von Hippel–Lindau protein (pVHL). To investigate whether effects of BAY 87-2243 on HIF target gene transcription are dependent on modulation of HIF protein expression, we incubated the renal cancer cell line RCC4 (which lacks functional pVHL and thus constitutively overexpresses HIF-1) with BAY 87-2243 under normoxic and hypoxic conditions. Western Blot analysis in cytosolic and nuclear extracts from normoxic H460 cells showed that BAY 87-2243 had no effect on HIF-1α protein levels in RCC4 cells (Fig. 4B). Real-time PCR-based quantification of the constitutively activated expression of HIF-1 target genes CA9, ANGPTL4, EGLN3, and EGLN2 mRNA levels revealed that BAY 87-2243 – even at concentrations up to 10 μmol/L – does not affect HIF-1-dependent gene expression in RCC4 cells (Fig. 4A). These findings exclude the possibility that BAY 87-2243 is an unspecific inhibitor of transcription and suggests that the activity on HIF and its downstream targets depends on a functional HIF degradation machinery which was further addressed with respect to PHD2.

### Silencing of PHD2 abolishes activity of BAY 87-2243 on the expression of HIF-1 target genes

It has been found that the specific silencing of PHD2 is sufficient for stabilizing HIF-1α and increasing its transcriptional activity^16^ although a recent publication indicated that PHD2 overexpression paradoxically results in a profound reduction of tumor growth, an effect, that was largely HIF-1α independent.^17^. To confirm that activity of BAY 87-2243 on HIF target gene transcription is dependent on EGLN1-mediated HIF-1α regulation, endogenous EGLN1 levels in normoxic H460 cells were lowered by siRNA-mediated gene silencing. In accordance with previous findings that silencing of EGLN1 augmented HIF-1α transcriptional activity and its target gene expression^16^, silencing of EGLN1 was found to be sufficient for stabilizing HIF-1α and increasing its transcriptional activity^18^. In line with this, the expression levels of HIF-1 target genes in H460 cells under normoxia are increased as revealed by real-time PCR (Fig. 4C). After confirming the efficacy of EGLN1-silencing by real-time PCR, H460 cells were incubated with 100 nmol/L BAY 87-2243. As expected, treatment lowered the low baseline (normoxic) expression levels of HIF-1 target genes CA9, EGLN3, and VEGF in nontransfected H460 cells and in H460 cells transfected with a scrambled siRNA control. In contrast, BAY 87-2243 had no effect on HIF-1 target gene expression levels in H460 cells silenced for EGLN1, further indicating that effects on HIF target gene transcription are dependent on an intact PHD–pVHL degradation pathway.

This was further confirmed by the finding that BAY 87-2243 had no effect on HIF-1 transcriptional activity in the presence of a PHDI: we incubated H460 cells under normoxia and hypoxia (16 h, 1% *p*O_2_) with various concentrations of BAY 87-2243 in the absence or presence of 5 μmol/L PHDI. Expression levels of HIF-1 target genes CA9, EGLN3, and ANGPTL4 and of EGLN2 was quantified by real-time PCR. As shown in Figure S2, BAY 87-2243 was inactive to suppress HIF-1 target genes under hypoxia when PHD activity was inhibited.

### BAY 87-2243 reduces tumor weight, HIF-1α protein, and HIF-1 target gene expression levels in H460 xenograft mouse model

**Figure 5:**
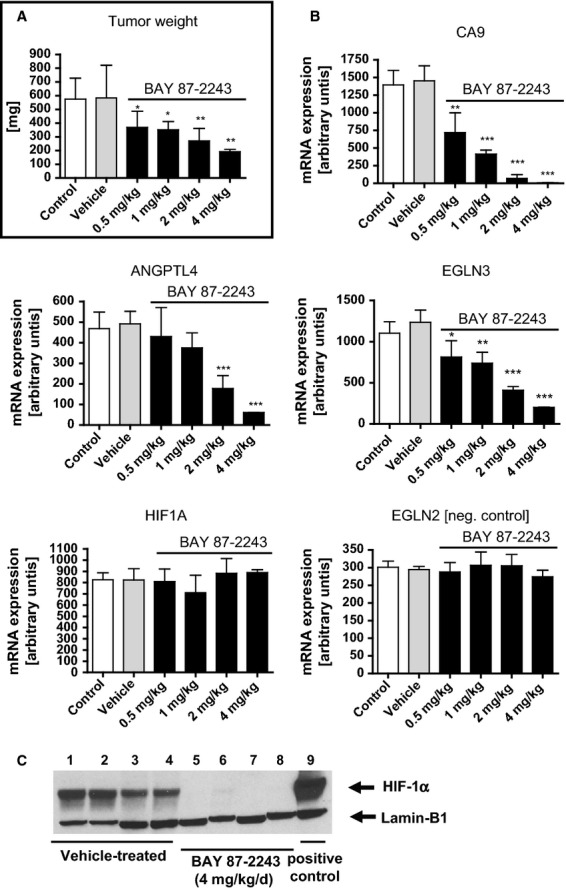
BAY 87-2243 reduces tumor weight, hypoxia-inducible factor (HIF)-1α protein levels, and HIF-1 target gene expression in H460 xenograft tumors. (A) Nude mice bearing established H460 human tumor xenografts were treated orally, once daily with BAY 87-2243 (0.5, 1.0, 2.0, and 4.0 mg/kg for 21 days and tumor weight was analyzed. (B) Total RNA from H460 xenograft tumors was isolated 16 h after last oral dosage of BAY 87-2243 at day 22 and the mRNA expression levels of HIF-1 target genes CA9, ANGPTL4, EGLN3, and of negative controls EGLN2 and HIF-1A was quantified by real-time PCR. (C) Nuclear extracts were isolated from four animals of the 4 mg/kg BAY 87-2243-treated group and from four animals from the vehicle-treated group. HIF-1α protein levels were assessed by Western blot analysis. A cell lysate from H460 cells incubated with 250 μmol/L CoCl_2_ for 16 h served as a positive control for the HIF-1α antibody. Lamin B1 served as a loading control.

Several small molecules that inhibit HIF-1α protein levels in vitro and in vivo have been described in the literature to reduce tumor burden in various mouse xenograft models, including deguelin^19^, aminoflavone^20^, and acriflavine^21^. Here, antitumor efficacy of BAY 87-2243 was evaluated in H460 xenograft mouse model. Nude mice were inoculated with H460 cells subcutaneously and after tumors have been established, animals were treated with BAY 87-2243 for 3 weeks by daily oral gavage. BAY 87-2243 reduced tumor weight dose dependently (Fig. 5A) in line with a dose-dependent reduction of the mRNA expression levels of the HIF-1 target genes CA9, ANGPTL4, and EGLN3, whereas the mRNA expression levels of hypoxia-insensitive EGLN2 gene and of HIF-1α itself were not affected by compound treatment in vivo (Fig. 5B). Tumor HIF-1α protein levels were undetectable by Western blot in BAY 87-2243-treated xenograft tumors in contrast to the vehicle-treated tumors (Fig. 5C). Plasma concentrations in each treatment group were determined 1 and 24 h after last oral dosing at day 21. Total plasma concentrations showed a direct pharmacodynamic relationship with the suppression of HIF-1 target gene expression in xenograft tumors (Table S3). With the exception of the 0.5 and 1 mg/kg dosage at 24 h after last treatment, plasma concentrations of free, unbound BAY 87-2243 are above the IC_50_ values calculated for the various pharmacodynamics effects, for example, inhibition of CA9 protein expression or inhibition of HIF reporter activity (see Fig. 1).

### BAY 87-2243 inhibits mitochondrial complex I activity

**Figure 6:**
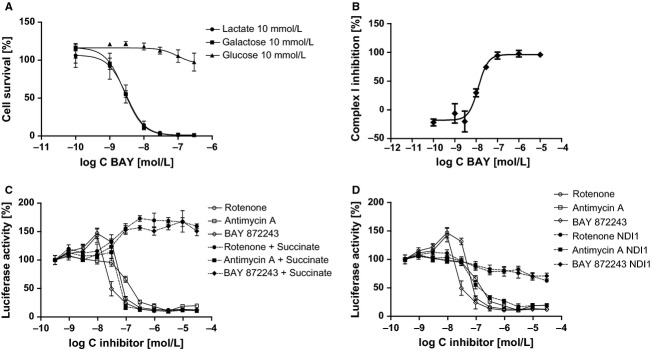
BAY 87-2243 inhibits mitochondrial complex I activity. (A) H460 cells were cultured in medium containing either glucose, galactose, or lactate as carbohydrate source and cell proliferation was measured by cell titer glow assay after 48 h of incubation with BAY 87-2243. (B) Inhibition of complex-I-dependent oxygen consumption in isolated mitochondria from PC3 cells by BAY 87-2243 was measured using LUX-MitoXpress system. (C) Luciferase activity was measured in H1299luc cells 1 h after incubation with either BAY 87-2243, rotenone, or antimycin A and a second measurement was performed after addition of 25 mmol/L succinate and 1 h further incubation. (D) Cellular ATP-dependent luciferase activity was measured in H1299luc cells and in H1299luc cells transfected with NDI1 after 1 h of incubation with either BAY 87-2243, rotenone, or antimycin A.

The ability to survive in media that are devoid of glucose but contain galactose is an indication of the mitochondrial function in cells. When cultured in galactose or lactate-containing media, cells depend solely on the oxidative phosphorylation in mitochondria for energy supply, which makes the cells highly sensitive to mitochondria inhibitors. In line with this, BAY 87-2243 had no effect on the proliferation of various human tumor cell lines when incubated in standard cell culture medium containing up to 10 mmol/L glucose (see Table S1). However, in galactose- or lactate-containing medium, proliferation, for example, of H460 cells was inhibited by BAY 87-2243 with an EC_50_ of ∼3 nmol/L, indicating that BAY 87-2243 is an inhibitor of mitochondrial function (Fig. 6A). To further confirm the involvement of mitochondrial respiratory chain in the HIF-1-inhibitory properties of BAY 87-2243, mitochondria were isolated from PC3 cells (containing more mitochondria than H460 cells) and incubated with the complex I substrates glutamate, malate, and ADP to stimulate maximal respiration rate (=100%) with or without complex I inhibitor rotenone (=0%). As shown in Figure 6B, BAY 87-2243 inhibits mitochondrial oxygen consumption measured by using the oxygen sensitive fluorescence dye LUX-MitoXpress with an IC_50_ value of ∼10 nmol/L.

Despite the well-established role of complex III in mitochondrial oxygen sensing^22^, more recent publications demonstrate that complex I activity limits the intracellular reactive oxygen species (ROS) production under low-oxygen conditions and that reducing mitochondrial complex I activity appears to be an essential element in the mitochondrial reprogramming induced by HIF-1^23^. Thus, we next examined if BAY 87-2243 activity is rather exerted by complex I or complex II or III inhibition. Inhibitors that selectively inhibit respiratory chain complex I can be identified by dual measurement of ATP-dependent luciferase activity in absence or presence of the complex II substrate succinate as described for oxygen consumption assays^24^. Therefore H1299luc cells were incubated with various concentrations of BAY 87-2243 under normoxic conditions in galactose-containing medium in the absence or presence of 25 mmol/L succinate. The decline of luciferase activity/cellular ATP content induced by either BAY 87-2243 or complex I inhibitor rotenone could be prevented by addition of complex II substrate succinate whereas the decline induced by complex III inhibitor antimycin A was not affected by succinate, indicating that BAY 87-2243 has no effect on complex III activity (Fig. 6C).

For additional verification that the decline of cellular ATP levels by BAY 87-2243-treatment is based on the inhibition of respiratory chain complex I, a rescue experiment using Nicotinamide adenine dinucleotide (NADH)-Q-Oxidoreductase from *S. cerevisiae* (NDI1) was performed. NDI1 is a monomeric enzyme with NADH-Q-Oxidoreductase activity that does not translocate protons and substitutes in yeast mitochondria the role of complex I. NDI1 can complement for dysfunctional complex I in mammalian cells^25^ and is insensitive to mammalian complex I inhibitors like rotenone^26^. H1299tluc cells were transfected with a pcDNA3 vector encoding for NDI1 under control of a CMV promoter and rotenone insensitive clones with high luminescence were used. Transfection of HT1299luc cells with NDI1 abolished the ATP decline observed in nontransfected HT1299luc cells after administration of either complex I inhibitor rotenone or BAY 87-2243 – but had no effect on ATP decline after treatment of Ht1299luc-NDI1 cells with complex III inhibitor antimycin A (Fig. 6D).

## Discussion

Inhibition of HIF-1 has emerged over the last several years as an attractive target for the development of novel cancer therapeutics. A variety of different approaches were followed to reduce HIF-1 activity in tumors, including antisense technology, for example, EZN-2968^27^ and small molecules that affect either HIF-1α translation, HIF-1α degradation, HIF-1α transcriptional activity, HIF-1 DNA binding, or HIF-1 dimerization under hypoxia^28^. Most of these compounds affect HIF-1 target gene expression only at micromolar concentrations like PX-478^29^ and acriflavine^21^ or display distinct pharmacological profiles with primary modes of action different from direct HIF-1 inhibition that may rather explain their antitumor efficacy in preclinical cancer models, for example, topoisomerase I inhibitors^30^, HSP90 inhibitors^31^, and Pi3K/Akt/mTOR inhibitors^19^,^32^. An excellent overview about the various different HIF-1 inhibitors is given in a recent review^33^.

This report describes the profile of an aminoalkyl pyrimidine that was identified as a highly potent inhibitor of HIF-1 activation in vitro and in vivo. BAY 87-2243 suppressed hypoxia-induced HIF target gene expression in vitro at low nanomolar concentrations but had only a weak activity on HIF-1 target genes under normoxia in line with findings of a low constitutive HIF activity under normoxia. BAY 87-2243 had virtually no effect on the expression levels of genes not known to be HIF target genes or to be induced by hypoxia even at concentrations tested up to 10 μmol/L. This is in contrast, for example, to HIF-1 inhibitor PX-478 which displayed suppression of HIF target genes in vitro and in vivo but also suppressed genes that are not known to be regulated by HIF suggesting that PX-478 does exhibit some nonspecific effects^3^. In contrast to PX-478, BAY 87-2243 did not affect HIF1α mRNA levels in vitro and in vivo.

Instead, BAY 87-2243 inhibited HIF-1α and HIF-2α protein accumulation in lung cancer cell line H460 under hypoxic conditions but had no effect on HIF-1α protein levels in H460 cells induced by the hypoxia mimetics desferrioxamine or cobalt chloride, indicating that PHD activity is essential for compound effect on HIF-1α. Furthermore, a possible increase of PHD activity upon BAY 87-2243 treatment could also be excluded as the underlying cause for the HIF-1α protein reduction, as the compound was inactive in a biochemical PHD activity assay. Given the importance of the axis “PHDs – VHL – proteasome” in the degradation of HIF-1α protein, we aimed to demonstrate if BAY 87-2243 activity is mediated upstream of PHD. In RCC4 cells lacking functional VHL protein, BAY 87-2243 did not affect HIF-1α protein levels and the siRNA-mediated inhibition of the HIF-1α-degrading PHD EGLN1 in H460 cells abolished activity of BAY 87-2243 on HIF-1 target genes. Furthermore, BAY 87-2243 did not suppress HIF-1 target gene expression under hypoxia if HIF-1α-degrading PHD were inhibited by a small molecule PHDI. Taken together, this data indicates that BAY 87-2243 activity is clearly dependent on functional PHD and VHL proteins.

In accordance with the high in vitro potency of BAY 87-2243 on the inhibition of HIF-1 target gene expression, the compound revealed antitumor efficacy in H460 xenograft model already at concentrations >0.5 mg/kg. Determination of BAY 87-2243 concentration in H460 xenograft plasma confirmed that the degree of suppression of HIF-1 target gene expression in xenograft tumors correlates with exposure and indicates that tumor weight reduction is a direct result of HIF-1 pathway inhibition in vivo. This data was further confirmed by the undetectable HIF-1α protein levels in BAY 87-2243-treated H460 xenograft tumors compared with vehicle-treated tumors which revealed a strong HIF-1α immunostaining in nuclear extracts. No signs of acute toxicity, for example, body weight loss or skin alterations were observed in mice. A potent inhibition of HIF-1α protein accumulation and suppression of HIF-1 target gene expression accompanied by in vivo antitumor efficacy has also been described for the naturally occurring rotenoid deguelin. However, this compound is an equipotent inhibitor of PI3K/Akt kinase activity, making it difficult to judge which proportion of the in vivo efficacy is attributable to HIF-1 inhibition. To the best of our knowledge, BAY 87-2243 does not affect other molecular targets that may easily explain the antitumor efficacy: BAY 87-2243 was tested against 221 kinases at a concentration of 10 μmol/L (Kinase Profiler, Millipore) and did not produce any hits. MDS (Ricerca) Lead Profiling Screen including a broad panel of receptors, ion channels, and transporters did not reveal significant binding of BAY 87-2243 to targets at relevant concentrations (<1 μmol/L).

Small molecule HIF-1 inhibitors from a class of alkyliminophenylacetate compounds that inhibit hypoxia-induced HIF-1 reporter activity at single-digit nanomolar concentrations have been described recently^9^. Further experiments revealed that all HIF-1 inhibitors from this chemical class exerted their effect on HIF-1 pathway through blocking the hypoxia-induced mitochondrial ROS production (data not shown). ROS have been found to inactivate HIF1α-degrading PHD and thereby increase HIF-1α levels highlighting the essential role of mitochondria as an oxygen sensor upstream of PHDs^34^. However, it remains to be determined whether the alkyliminophenylacetate class of compounds could be tolerated in animal models at dosages that inhibit HIF-1 in vivo and display antitumor efficacy.

We carried out several different experiments to analyze BAY 87-2243 effect on mitochondrial function. First, we demonstrated that BAY 87-2243 potently inhibits H460 cell proliferation under conditions favoring mitochondrial oxidative phosphorylation as energy source and thus making cells highly sensitive to mitochondria inhibitors as described for alkyliminophenylacetate compounds. Second, we demonstrated that BAY 87-2243 inhibits oxygen consumption of isolated mitochondria from PC3 cells; an effect that was completely abolished by an excess of complex I inhibitor rotenone. Finally, we demonstrated that either the addition of complex II substrate succinate or the rescue of complex I inhibition phenotype by complementation with rotenone insensitive NADH-Q-oxidoreductase from *S. cerevisiae* prevented cytotoxicity of BAY 87-2243 on H1299 cells. Taken together, these results are all in line with an inhibition of mitochondrial complex I activity by BAY 87-2243. This, in turn, prevents the ROS-mediated inhibition of HIF-1 degrading PHD and thus reduces HIF-1α protein levels under hypoxic conditions. The mode-of-action of BAY 87-2243 can also be regarded as a restorage of PHD activity and this explains why compound activity strictly depends on functional PHD/pVHL proteins. In contrast to complex-I-inhibitor rotenone, BAY 87-2243 displayed no cytotoxicity to human dopaminergic neuroblastoma cell line SH-SY5Y under normoxia (see Fig. S3), an established cell culture model of Parkinson's disease^35^, supporting recent findings that neuronal toxicity of rotenone is rather mediated by mechanisms other than inhibition of mitochondrial basal oxygen consumption rate, for example, microtubule destabilization via Glaxo Smithkline3β activation^36^. As it has become more and more accepted that targeting mitochondrial proteins by small molecule compounds might be an attractive therapeutic option to overcome some forms of drug resistance, as recently reviewed in^38^,^37^,^39^, the contribution of complex I inhibitors such as BAY 87-2243 to this emerging field remains to be evaluated in the near future.

## Supporting Information

**Table S1.** BAY 87-2243 has no effect on cell proliferation in vitro. The CellTiter Glo kit (Promega) was used to assess cytotoxicity. BAY 87-2243 was added 24 h after seeding; the CellTiter Glo assay was performed after 48 h of incubation. The IC50 value was calculated with Graph- Pad Prism software using four parameter logistic equation. Table S2. Primer sequences used for real-time PCR. Table S3. Plasma concentrations of BAY 87-2243 in H460 xenografted NMRI nude mice at 1 and 24 h after last oral dosing. **Figure S1.** BAY 87-2243 has no effect on mRNA levels of genes that are insensitive to hypoxia. A549 cells were incubated with up to 10 μmol/L BAY 87-2243 for 16 h under either normoxia (white bars) or hypoxia (1% pO2). RNA was isolated and the expression of the housekeeping genes cytosolic beta actin (ACTB), beta-2 microglobulin (B2M), and the transcriptions factors forkhead box O3A (FOXO3A) and GATA binding protein 4 (GATA4 were quantified by real-time PCR. **Figure S2.** BAY 87-2243 is inactive in the presence of a prolyl hydroxylase inhibitor (PHDI). A549 cells were incubated for 16 h with the indicated concentrations of BAY 87-2243 in either normoxia or hypoxia (1% pO2). Expression levels of HIF-1 target genes CA9, EGLN3 and ANGPTL4 and of negative control EGLN2 was quantified by real-time PCR. **Figure S3.** Complex-I-inhibitor rotenone, but not BAY 87-2243 is cytotoxic to the human dopaminergic neuroblastoma cell line SH-SY5Y. SH-SY5Y cells were cultured under normoxic conditions in medium containing normal glucose levels and cell viability was measured by cell titer glow assay after 72 h of incubation with either BAY 87-2243 or rotenone.
