# Supplementary material for: BAY 87-2243, a highly potent and selective inhibitor of hypoxia-induced gene activation has antitumor activities by inhibition of mitochondrial complex I
Source: Cancer Med. 2013 Aug 20;2(5):611–24. doi: 10.1002/cam4.112 (PMC3892793; doi:10.1002/cam4.112)
Supplement: Supplementary file 1 — Table S1. BAY 87-2243 has no effect on cell proliferation in vitro. The CellTiter Glo kit (Promega) was used to assess cytotoxicity. BAY 87-2243 was added 24 h after seeding; the CellTiter Glo assay was performed after 48 h of incubation. The IC50 value was calculated with Graph- Pad Prism software using four parameter logistic equation. Table S2. Primer sequences used for real-time PCR. Table S3. Plasma concentrations of BAY 87-2243 in H460 xenografted NMRI nude mice at 1 and 24 h after last oral dosing. Figure S1. BAY 87-2243 has no effect on mRNA levels of genes that are insensitive to hypoxia. A549 cells were incubated with up to 10 μmol/L BAY 87-2243 for 16 h under either normoxia (white bars) or hypoxia (1% pO2). RNA was isolated and the expression of the housekeeping genes cytosolic beta actin (ACTB), beta-2 microglobulin (B2M), and the transcriptions factors forkhead box O3A (FOXO3A) and GATA binding protein 4 (GATA4 were quantified by real-time PCR. Figure S2. BAY 87-2243 is inactive in the presence of a prolyl hydroxylase inhibitor (PHDI). A549 cells were incubated for 16 h with the indicated concentrations of BAY 87-2243 in either normoxia or hypoxia (1% pO2). Expression levels of HIF-1 target genes CA9, EGLN3 and ANGPTL4 and of negative control EGLN2 was quantified by real-time PCR. Figure S3. Complex-I-inhibitor rotenone, but not BAY 87-2243 is cytotoxic to the human dopaminergic neuroblastoma cell line SH-SY5Y. SH-SY5Y cells were cultured under normoxic conditions in medium containing normal glucose levels and cell viability was measured by cell titer glow assay after 72 h of incubation with either BAY 87-2243 or rotenone. [file cam40002-0611-sd1.doc]

**Supporting information**

**
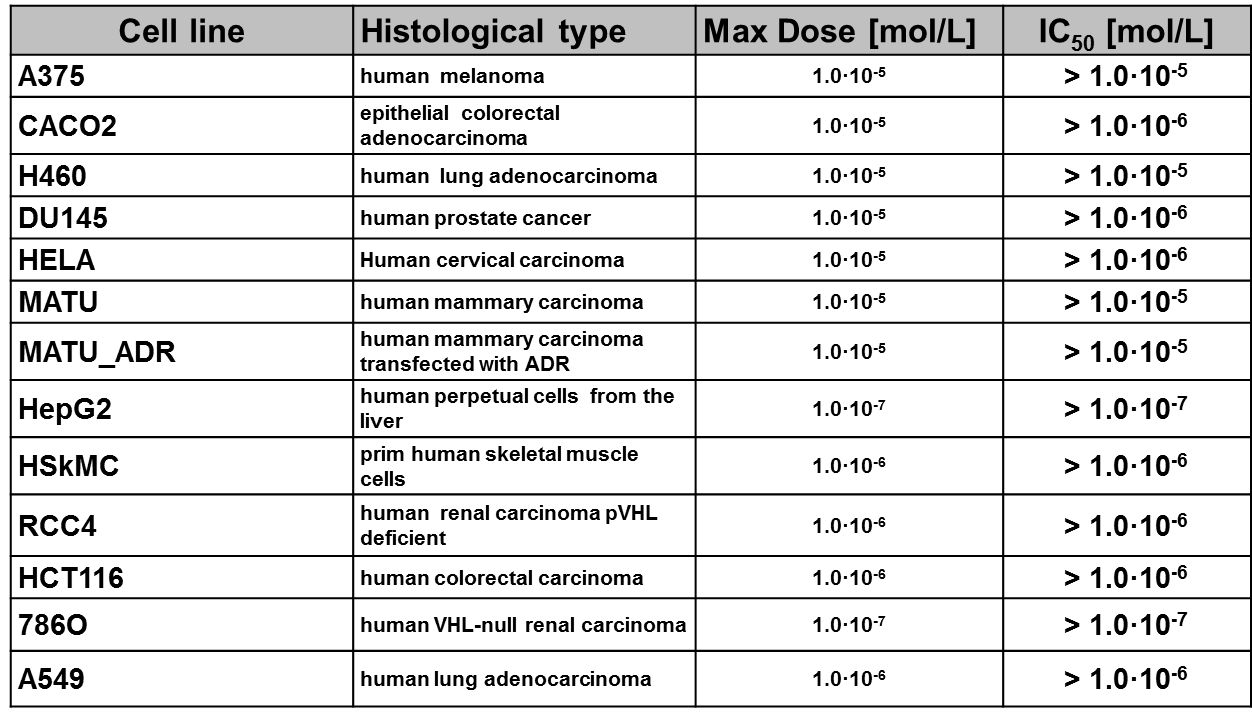
**

**Table S1:** BAY 87-2243 has no effect on cell proliferation *in vitro.* The CellTiter Glo kit (Promega) was used to assess cytotoxicity. BAY 87-2243 was added 24 h after seeding; the CellTiter Glo assay was performed after 48 hrs of incubation. The IC50 value was calculated with GraphPad Prism software using four parameter logistic equation.

**Table S2**: Primer sequences used for real-time PCR

**Table S3**: Plasma concentrations of BAY 87-2243 in H460 xenografted NMRI nude mice at 1 h and 24 h after last oral dosing

**Figure S1**: BAY 87-2243 has no effect on mRNA levels of genes that are insensitive to hypoxia. A549 cells were incubated with up to 10 µM BAY 87-2243 for 16 h under either normoxia (white bars) or hypoxia (1 % pO_2_). RNA was isolated and the expression of the housekeeping genes cytosolic beta actin (ACTB), beta-2 microglobulin (B2M), and the transcriptions factors forkhead box O3A (FOXO3A) and GATA binding protein 4 (GATA4were quantified by real-time PCR.

**Figure S2:** BAY 87-2243 is inactive in the presence of a prolyl hydroxylase inhibitor (PHDI). A549 cells were incubated for 16 h with the indicated concentrations of BAY 87-2243 in either normoxia or hypoxia (1 % pO_2_). Expression levels of HIF-1 target genes CA9, EGLN3 and ANGPTL4 and of negative control EGLN2 was quantified by real-time PCR.

**Figure S3**: Complex-I-inhibitor rotenone, but not BAY 87-2243 is cytotoxic to the human dopaminergic neuroblastoma cell line SH-SY5Y. SH-SY5Y cells were cultured under normoxic conditions in medium containing normal glucose levels and cell viability was measured by cell titer glow assay after 72 hours of incubation with either BAY 87-2243 or rotenone.
